# Efficacy of octreotide long-acting repeatable in neuroendocrine tumors: RADIANT-2 placebo arm *post hoc* analysis

**DOI:** 10.1530/ERC-15-0314

**Published:** 2015-12

**Authors:** Jonathan R Strosberg, James C Yao, Emilio Bajetta, Mounir Aout, Bert Bakker, John D Hainsworth, Philippe B Ruszniewski, Eric Van Cutsem, Kjell Öberg, Marianne E Pavel

**Affiliations:** 1 Department of Medicine, Moffitt Cancer Center, Tampa, Florida, USA; 2 Division of Cancer Medicine, Department of Gastrointestinal Medical Oncology, The University of Texas MD Anderson Cancer Center, Houston, Texas, USA; 3 Istituto di Oncologia, Policlinico di Monza, Monza, Italy; 4 Novartis International AG, Basel, Switzerland; 5 Novartis Pharmaceuticals Corporation, East Hanover, New Jersey, USA; 6 Sarah Cannon Research Institute, Nashville, Tennessee, USA; 7 University of Paris VII and Beaujon Hospital, Paris, France; 8 Digestive Oncology, University Hospitals Gasthuisberg, Leuven and KULeuven, Leuven, Belgium; 9 Uppsala University Hospital, Uppsala, Sweden; 10 Department of Hepatology and Gastroenterology, Charité-Universitätsmedizin Berlin, Campus Virchow Klinikum, Berlin, Germany

**Keywords:** neuroendocrine tumors, octreotide LAR, progression-free survival, somatostatin analogues

## Abstract

Somatostatin analogues (SSA) have demonstrated antiproliferative activity in addition to efficacy for carcinoid symptom control in functional neuroendocrine tumors (NET). A *post hoc* analysis of the placebo arm of the RAD001 In Advanced Neuroendocrine Tumors-2 (RADIANT-2) study was conducted to assess the efficacy of octreotide long-acting repeatable (LAR) on progression-free survival (PFS) and overall survival (OS) estimated using the Kaplan–Meier method. Out of 213 patients randomized to placebo plus octreotide LAR in RADIANT-2, 196 patients with foregut, midgut, or hindgut NET were considered for present analysis. Of these, 41 patients were SSA-treatment naïve and 155 had received SSA therapy before study entry. For SSA-naïve patients, median PFS by adjudicated central review was 13.6 (95% CI 8.2–22.7) months. For SSA-naïve patients with midgut NET (*n*=24), median PFS was 22.2 (95% CI 8.3–29.5) months. For patients who had received SSA previously, the median PFS was 11.1 (95% CI 8.4–14.3) months. Among the SSA-pretreated patients who had midgut NET (*n*=119), the median PFS was 12.0 (95% CI 8.4–19.3) months. Median OS was 35.8 (95% CI 32.5–48.9) months for patients in the placebo plus octreotide LAR arm; 50.6 (36.4 – not reached) months for SSA-naïve patients and 33.5 (95% CI 27.5–44.7) months for those who had received prior SSA. This *post hoc* analysis of the placebo arm of the large phase 3 RADIANT-2 study provides data on PFS and OS among patients with progressive NET treated with octreotide therapy.

## Introduction

Neuroendocrine tumors (NET) are a rare, heterogeneous group of tumors that arise from neuroendocrine cells throughout the body and are capable of producing various bioactive substances such as peptides and amines (Yao *et al*. 2008, Vinik *et al*. 2010). Somatostatin analogues (SSA) can effectively suppress the secretion of serotonin and other bioactive substances and relieve associated symptoms in patients with functional NET. In addition to symptom control, therapeutic management for patients with advanced, unresectable NET aim to induce tumor regression or stabilization and improve survival (Pavel *et al*. 2012). Octreotide and lanreotide have demonstrated anti-proliferative activity in pivotal phase 3 trials (Rinke *et al*. 2009, Caplin *et al*. 2014*a*).

In the landmark PROMID (Placebo-controlled, double-blind, prospective, Randomized study of the effect of octreotide long-acting repeatable (LAR) in the control of tumor growth in patients with metastatic neuroendocrine midgut tumors; *n*=85) study, octreotide LAR (30 mg intramuscularly every 28 days) demonstrated antitumor activity in patients with newly diagnosed advanced metastatic NET of midgut origin. The median time to tumor progression was 14.3 months with octreotide LAR vs 6.0 months with placebo (hazard ratio (HR), 0.34; 95% CI 0.20–0.59; *P*=0.000072) using the World Health Organization (WHO) response criteria for tumor evaluation (Rinke *et al*. 2009). In the more recent phase 3 CLARINET study (*n*=204), patients with advanced NET treated with lanreotide autogel (120 mg via deep s.c. injection every 28 days) also had improvement in progression-free survival (PFS) when compared with placebo (PFS, not reached (NR) vs 18 months and HR, 0.47; 95% Cl 0.30–0.73; *P*<0.001) (Caplin *et al*. 2014*a*).

In the large, phase 3 RADIANT-2 (RAD001 In Advanced Neuroendocrine Tumors-2, Second Trial) study, everolimus in combination with octreotide LAR showed a clinically meaningful 5.1-month increase in median PFS, compared with placebo plus octreotide LAR treatment in patients with advanced NET associated with carcinoid syndrome, although the study did not meet its pre-specified endpoint (median PFS, 16.4 vs 11.3 months and HR, 0.77; 95% CI 0.59–1.00; *P*=0.026, pre-specified *P*=0.0246; Pavel *et al*. 2011).

This exploratory *post hoc* analysis of the placebo plus octreotide LAR arm of the RADIANT-2 study was conducted to assess the effect of octreotide LAR on survival endpoints in a heterogeneous group of patients with advanced NET.

## Patients and methods

### Study design

RADIANT-2 was a multinational, multicenter, randomized, double-blind, placebo-controlled phase 3 study (ClinicalTrials.gov number, NCT00412061; http://clinicaltrials.gov/show/NCT00412061); the detailed study design has been described earlier (Pavel *et al*. 2011). Patients were randomly assigned (1:1) to receive everolimus (10 mg/day) plus octreotide LAR (30 mg intramuscularly every 28 days) or placebo plus octreotide LAR (30 mg intramuscularly every 28 days). The study treatment continued until disease progression, development of unacceptable adverse events, withdrawal of patient consent, or primary analysis (cutoff date, 2 April 2010). During the double-blind phase, patients were permitted to crossover from the placebo plus octreotide LAR arm to the open-label everolimus plus octreotide LAR arm upon disease progression according to the Response Evaluation Criteria in Solid Tumors (RECIST, version 1.0; Therasse *et al*. 2000). A blind was broken for these patients.

At the end of the double-blind phase, all ongoing patients were unblinded and were allowed to transition to open-label everolimus plus octreotide LAR at the discretion of the treating physician. All patients who switched to the open-label everolimus plus octreotide LAR (during the double-blind phase or after unblinding) discontinued everolimus treatment upon disease progression. These patients were then followed up monthly for survival information (until 13 June 2013, data cutoff date for the overall survival (OS) analysis).

**Figure 1:**
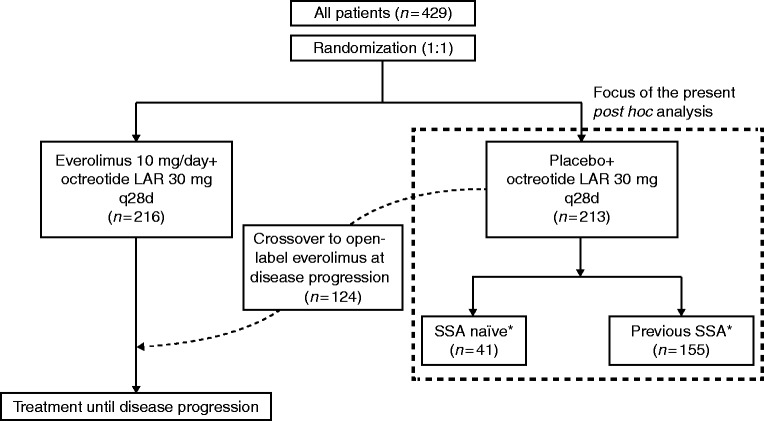
RADIANT-2 study design and the focus of the present *post hoc* analysis. LAR, long-acting repeatable; q28d, every 28 days; SSA, somatostatin analogues. *A total of 196 patients (41 SSA-naïve and 155 who had received SSA previously) had foregut, midgut, or hindgut NET and were included in the present analysis.

The patients enrolled in the placebo plus octreotide LAR arm were the focus of the present *post hoc* analysis (Fig. 1).

### Ethics

The study protocol and all amendments were reviewed and approved by independent ethics committees or Institutional Review Boards for all participating centers. The study conformed to the Good Clinical Practice guidelines and was conducted in the spirit of the principles set in the Declaration of Helsinki. Written informed consent was obtained from all patients prior to participation.

### Patient population

The patient population has been described earlier in detail (Pavel *et al*. 2011). Briefly, adult patients (age ≥18 years) with low- or intermediate-grade advanced (unresectable locally advanced or distant metastatic) NET, a history of symptoms attributed to carcinoid syndrome (flushing, diarrhea, or both) and disease progression within the past 12 months were eligible. Other key inclusion criteria included the presence of measurable disease according to RECIST, version 1.0 (Therasse *et al*. 2000), and a WHO performance status (PS) score of ≤2 with adequate bone marrow, liver, and kidney function.

Patients with poorly differentiated or high-grade NET were not eligible. Furthermore, patients were ineligible if they had received cytotoxic chemotherapy, immunotherapy, or radiotherapy within 4 weeks prior to randomization, or prior therapy with mammalian target of rapamycin inhibitors (sirolimus, temsirolimus, or everolimus). Patients who had received SSA treatment within 2 weeks prior to randomization were ineligible. Information on the previous SSA dose was not available for all patients.

Patients with foregut, midgut, or hindgut NET were considered for this subgroup analysis of placebo plus octreotide LAR arm of the RADIANT-2 study. Tumors originating in the lung, stomach, duodenum, or pancreas were categorized as foregut NET; tumors originating in the small intestine, appendix, proximal colon, or of unknown primary sites were considered to be midgut NET; and tumors with the colon (transverse or distal) or rectum as the primary site were classified as hindgut NET.

### Statistical analysis

In the primary analysis of the RADIANT-2 study, the primary endpoint was PFS, assessed by an adjudicated central radiology review according to RECIST, version 1.0 (Pavel *et al*. 2011). PFS (data cutoff date, 2 April 2010) was estimated using the Kaplan–Meier method and reported with corresponding 95% CIs. The median PFS was also assessed by local investigators. The secondary endpoint was OS (data cutoff date, 13 June 2013).

For this *post hoc* analysis, patients were stratified based on previous SSA use and tumor location (foregut, midgut, or hindgut), and the median PFS and OS were calculated in these subgroups.

In a multivariate analysis, HRs and 95% CIs were calculated with a Cox proportional hazards model using specified variables selected by stepwise regression. The specified variables included age (<65 or ≥65 years), sex, race (Caucasian or non-Caucasian), WHO PS score (0 or >0), previous octreotide use, baseline chromogranin A level (≤2× upper limit of normal (ULN) or >2× ULN), baseline 5-hydroxyindoleacetic acid level (elevated (greater than median) or normal (less than or equal to median)), and primary tumor site.

## Results

### Patient demographics

Among 213 patients assigned to the placebo plus octreotide LAR arm, 196 patients had foregut, midgut, or hindgut NET and were included in the present analysis. Of these, 155 (79%) patients had received previous SSA therapy (previous SSA group), whereas 41 (21%) had not (SSA-naïve group; Table 1).

The time since initial diagnosis was longer for patients who received previous SSA therapy; 7% of patients in the previous SSA group had a diagnosis of NET within 6 months prior to study entry, compared with 24% of patients in the SSA-naïve group.

All patients qualifying for enrollment in the RADIANT-2 study had progressive disease within the past 12 months and history of carcinoid symptoms (diarrhea or flushing). For the 155 patients who had received SSA therapy prior to study entry, the median exposure to prior SSA was 1.8 (range 0–12.5) years. Of these 155 patients, 142 (92%) received octreotide LAR and the remaining 13 (8%) patients received lanreotide. Seventy-three (51%) of the 142 patients on previous octreotide received a dose of ≥30 mg every 4 weeks and 68 (48%) patients received a dose <30 mg every 4 weeks; for one patient, information on the prior octreotide dose was missing.

### Progression-free survival

**Figure 2:**
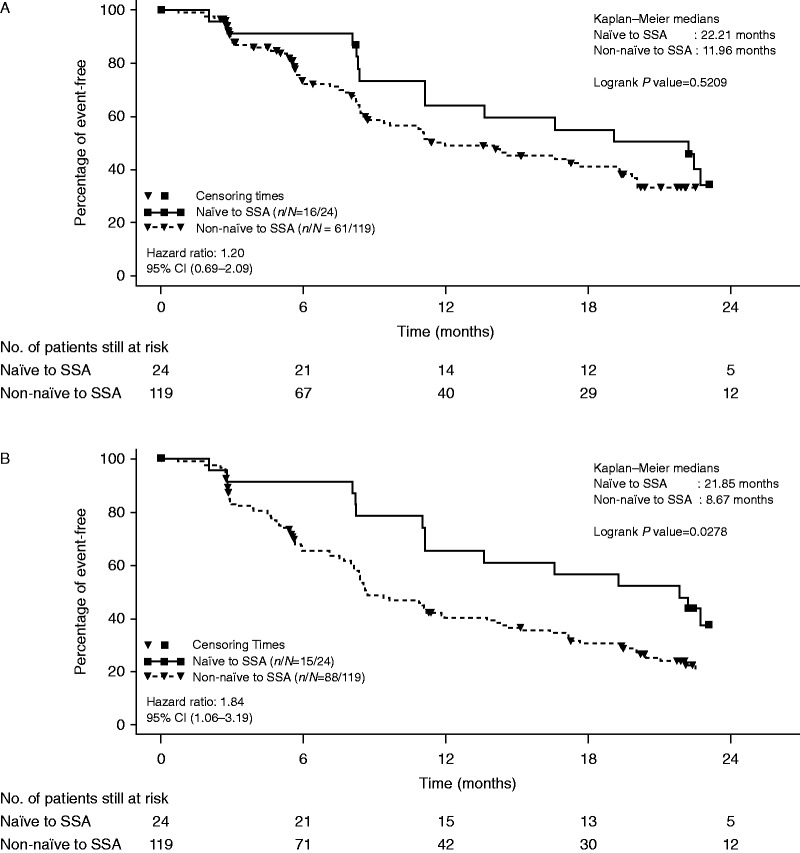
Kaplan–Meier plots of progression-free events in patients with midgut tumors. (A) By adjudicated central review. (B) By local assessment. SSA, somatostatin analogues.

Based on the findings of the adjudicated central assessment for the placebo plus octreotide LAR arm, the median PFS was 13.6 (95% CI 8.2–22.7) months for SSA-naïve patients and 11.1 (95% CI 8.4–14.3) months for patients who had received SSA therapy previously. Among patients with midgut NET, the median PFS was 22.2 (95% CI 8.3–29.5) months for SSA-naïve patients, and 12.0 (95% CI 8.4–19.3) months for patients who had prior exposure to SSA. Figure 2 illustrates the median PFS in patients with midgut tumors by prior SSA use as per local investigator and per adjudicated central review.

Multivariate analysis for PFS showed that baseline WHO PS of >0 (HR (95% CI), 0.65 (0.44–0.98), *P*=0.038) was associated with decreased PFS, and non-elevated baseline CgA level (HR (95% CI), 2.26 (1.45–3.52), *P*<0.001) were associated with increased PFS.

### Overall survival

For patients in the placebo plus octreotide LAR arm of RADIANT-2, the median OS was 35.8 (95% CI 32.5–48.9) months. For SSA-naïve patients, the median OS was 50.6 (95% CI, lower limit, 36.4; upper limit, NR) months, compared with 33.5 (95% CI 27.5–44.7) months for patients who had received previous SSA therapy (Table 2).

In multivariate analysis for survival, baseline WHO PS of >0 (HR (95% CI), 0.39 (0.26–0.58), *P*<0.001) was associated with a decreased in OS; and non-elevated baseline CgA level (HR (95% CI), 2.08 (1.21–3.60), *P*=0.009), and non-elevated 5-hydroxyindoleacetic acid level (HR (95% CI), 1.69 (1.07–2.69), *P*=0.026) were found to be prognostic of increased OS.

## Discussion

In addition to antisecretory efficacy, SSA have demonstrated antitumor activity in the phase 3 PROMID and CLARINET studies. The PROMID study evaluated octreotide LAR vs placebo in treatment-naïve, low-grade midgut NET, whereas the CLARINET study evaluated lanreotide autogel vs placebo in a more heterogeneous population of enteropancreatic NET, nearly all of whom had stable disease at the outset (Rinke *et al*. 2009, Caplin *et al*. 2014*a*). In contrast, the present *post hoc* analysis of the placebo plus octreotide LAR arm of the RADIANT-2 study provides the outcomes of patients with progressive NET who were started on long-acting SSA treatment.

The CLARINET study allowed patients from the placebo arm to receive lanreotide autogel in an open-label extension phase, upon disease progression during the double-blind core phase. Data from the open-label extension phase of the CLARINET study demonstrated a median PFS of 14.0 months after switch to lanreotide autogel (Caplin *et al*. 2014*b*). Of note, despite differences in the patient population between the RADIANT-2 and CLARINET studies (Pavel *et al*. 2011, Caplin *et al*. 2014*a*) the median PFS observed in the open-label extension phase of the CLARINET study is similar to that reported in the present analysis.

The results of this analysis suggest an expected clinical benefit of octreotide treatment for SSA-naïve patients with midgut NET. The data indicated that the median PFS was 22.2 (95% CI 8.3–29.5) months among patients with midgut NET who were SSA-naïve and went on to receive octreotide LAR on the trial. For patients with midgut NET who had been treated with SSA previously, the median PFS was 13.6 (95% CI 8.2–22.7) months.

The findings furthermore provide a clearer picture regarding PFS by RECIST criteria in patients with midgut NET treated with SSA. In the PROMID study, the median time to tumor progression in the octreotide LAR arm was 14.3 months, whereas in the CLARINET study, the median PFS was NR in the lanreotide autogel arm after a median follow-up of 27 months (Rinke *et al*. 2009, Caplin *et al*. 2014*a*). A potential explanation for this large disparity in PFS is the differing criteria for progression in the two studies: WHO criteria in PROMID (25% increase in two dimensions) and RECIST criteria in CLARINET (20% increase in one dimension, which corresponds to a 44% increase in two dimensions) (James *et al*. 1999, Therasse *et al*. 2005, Oxnard *et al*. 2012). In our present analysis, which used RECIST criteria, the median PFS was 22 months among SSA-naïve patients with midgut NET, all of whom were progressive prior to study entry. This finding from the present subgroup analysis is also aligned with CLARINET data. It is therefore increasingly apparent that the median time to tumor progression of 14.3 months in the PROMID study is an underestimation of PFS by RECIST criteria. In other words, when patients with midgut NET are treated with SSA therapy, one can expect a median time to progression of ∼2 years by RECIST criteria.

The results for the final OS analysis of the RADIANT-2 study placebo arm demonstrate longer survival in SSA-naïve patients treated with octreotide LAR. The median OS was 50.6 months among SSA-naïve patients compared with 33.5 months for patients who had received prior SSA treatment. Moreover, among SSA-naïve patients with midgut NET, the median OS was NR with a median follow-up of 64 months. Notably, in the SSA-naïve group, the proportion of patients with longer duration since initial diagnosis was lower than in the group with previous SSA therapy. It is likely that the shorter duration since diagnosis in the SSA-naïve group may have resulted in a longer OS. The complete results for the secondary endpoint of OS will be reported separately.

A major limitation of this analysis is the fact that only 41 patients on the octreotide plus placebo arm of the RADIANT-2 study were SSA-naïve. As our analysis focuses on this subset of patients, all median PFS and OS figures are associated with large CIs, limiting the reliability of these estimates. The findings warrant further confirmation from larger prospective studies.

## Conclusions

The *post hoc* analysis of the placebo arm of large phase 3 RADIANT-2 study, for the first time, provides data on PFS and OS from a prospective study among patients with progressive NET treated with octreotide therapy. This *post hoc* analysis showed that SSA-naïve patients with progressive midgut NET treated with octreotide LAR had a relatively long median PFS of 22.2 months, exceeding the time to tumor progression of 14.3 months observed in the PROMID study. The median PFS assessed by RECIST criteria may represent a more accurate reflection of the true PFS associated with SSA use in midgut NET.

## Earlier presentations

Presented at the Gastrointestinal Cancers Symposium (ASCO GI), January 16–18, 2014, San Francisco, California, and at the European Neuroendocrine Tumor Society (ENETS), March 5–7, 2014, Barcelona, Spain.

## Author contribution statement

E Van Cutsem, J C Yao, and K Öberg performed the study design. E Van Cutsem, J C Yao, J R Strosberg, M E Pavel, and P B Ruszniewski were involved in the recruitment of patients. M Aout supported statistical analysis and provided results and data interpretation. J D Hainsworth also contributed to statistical analysis. E Bajetta, J C Yao, J R Strosberg, and M E Pavel were involved in data interpretation. B Bakker, J C Yao, J D Hainsworth, and J R Strosberg contributed to writing of the manuscript content. B Bakker, E Bajetta, E Van Cutsem, J C Yao, J D Hainsworth, J R Strosberg, K Öberg, M Aout, P B Ruszniewski, and M E Pavel critically revised the content and approved the final draft of the manuscript for publication.

## Figures and Tables

**Table 1:** Baseline demographics and clinical characteristics (stratified by previous SSA use)

**Characteristic**	**SSA naïve** (*n*=41)	**Previous SSA** (*n*=155)	**All patients** (*n*=196)
Median age (range), years	59 (37–75)	60 (27–81)	60 (27–81)
Sex, *n* (%)			
Male	22 (54)	92 (59)	114 (58)
Female	19 (46)	63 (41)	82 (42)
WHO PS, *n* (%)^a^			
0	30 (73)	100 (65)	130 (66)
1	11 (27)	45 (29)	56 (29)
2	0	10 (6)	10 (5)
Tumor location, *n* (%)^b^			
Foregut	15 (37)	17 (11)	32 (16)
Midgut	24 (59)	119 (77)	143 (73)
Hindgut	2 (5)	19 (12)	21 (11)
Histology, *n* (%)^c^			
Well differentiated or low-grade	32 (78)	128 (83)	160 (82)
Moderately differentiated or intermediate-grade	9 (22)	20 (13)	29 (15)
Poorly differentiated or high-grade	0	1 (<1)	1 (<1)
Unknown	0	6 (4)	6 (3)
Time since initial diagnosis, *n* (%)^d^			
≤6 months	10 (24)	11 (7)	21 (11)
>6 months–≤2 years	9 (22)	37 (24)	46 (23)
>2–≤5 years	14 (34)	35 (23)	49 (25)
>5–≤10 years	6 (15)	51 (33)	57 (29)
>10 years	2 (5)	20 (13)	22 (11)
Systemic antitumor drugs, *n* (%)			
Chemotherapy	11 (27)	43 (28)	54 (28)
Immunotherapy	1 (2)	18 (12)	19 (10)
Targeted therapy	1 (2)	13 (8)	14 (7)
Other	1 (2)	25 (16)	26 (13)
Previous SSA therapy, *n* (%)			
Octreotide LAR	–	142 (92)	142 (72)^e^
Lanreotide	–	13 (8)	13 (7)^e^

WHO PS, World Health Organization performance status; SSA, somatostatin analogues; LAR, long-acting repeatable.

a Data missing for one patient.

b Tumors originating in the lung, stomach, duodenum, or pancreas were categorized as foregut NET; those originating in the small intestine, appendix, proximal colon, or of unknown primary sites were considered to be midgut NET; and those with the colon (transverse or distal) or rectum as the primary site were classified as hindgut NET.

c The histology-based categories as reported in pathology reports. At the time of study conduct, the harmonized definition of grading for NET was not established and the WHO classification was not in practice. Thus, the categories reported here may not translate in to strict numerical categories based on WHO grading (based on mitotic count and/or Ki-67 labeling index) being used in current clinical practice.

d Data missing for two patients.

e Total is not 100%, as 41 (21%) patients did not receive prior SSA therapy.

**Table 2:** OS (data cutoff date, 13 June 2013)

**Patient population**	**SSA naïve**		**Previous SSA**^a^	
*n* (%)	Median OS, months (95% CI)	*n* (%)	Median OS, months (95% CI)
Total	41 (100)	50.6 (36.4–NR)	155 (100)	33.5 (27.5–44.7)
By tumor location				
Foregut	15 (37)	36.4 (25.5–NR)	17 (11)	33.6 (16.7–NR)
Midgut	24 (59)	NR (42.4–NR)	119 (77)	33.5 (27.5–49.4)
Hindgut	2 (5)	NR (4.7–NR)	19 (12)	35.8 (15.4–48.9)

SSA, somatostatin analogues; OS, overall survival; NR, not reached.

a Includes patients who had received previous octreotide or lanreotide treatment.
